# A New Era for Esketamine in Managing Treatment-Resistant Depression: A Systematic Review of Its Use From Adjunct to First-Line Therapy

**DOI:** 10.7759/cureus.91829

**Published:** 2025-09-08

**Authors:** Bhavya, Srijan Shetty, Balakrishnan Sadasivam

**Affiliations:** 1 Pharmacology, All India Institute of Medical Sciences, Bhopal, IND

**Keywords:** esketamine, esketamine monotherapy, nasal spray, trd, treatment-resistant depression (trd)

## Abstract

Treatment-resistant depression (TRD) remains a major clinical challenge, affecting nearly one-third of individuals with major depressive disorder (MDD) who fail to respond to standard antidepressant therapies. Esketamine, the S-enantiomer of ketamine, has emerged as a novel therapeutic option. Esketamine, initially introduced as an add-on therapy, has more recently also received recognition for use as a standalone treatment. This review aims to synthesize current clinical evidence on the efficacy and safety of esketamine monotherapy while exploring its molecular mechanisms and identifying research gaps. A systematic literature search was conducted across PubMed, Google Scholar, MedRxiv, and BioRxiv up to March 2025. Although designed as a systematic review, only one qualifying clinical trial was identified, limiting the feasibility of a meta-analysis and underscoring the scarcity of direct evidence. Despite this limitation, molecular insights suggest that TRD is associated with glutamatergic system dysfunction, impaired neuroplasticity, hypothalamic-pituitary-adrenal (HPA) axis abnormalities, and neuroinflammatory processes. Intranasal esketamine demonstrates potential as a rapid-acting and effective standalone therapy for TRD; nevertheless, further robust, large-scale randomized controlled trials are essential to confirm its therapeutic benefit, establish optimal dosing strategies, and overcome current research limitations.

## Introduction and background

Treatment-resistant depression (TRD) refers to major depressive disorder (MDD) in which an individual does not achieve an adequate response despite receiving at least two different antidepressant medications at appropriate doses and for a sufficient duration [1]. While there is no universally accepted definition of appropriate dosage and treatment duration, TRD is generally diagnosed when a patient shows less than a 50% improvement in depressive symptoms after four weeks of optimal treatment with at least two different prescribed antidepressants [2,3].

TRD is often reported to affect around 30% of individuals undergoing antidepressant treatment in research settings, whereas its prevalence in real-world practice is estimated to range between 6% and 55% [4]. MDD is among the most prevalent psychiatric conditions, affecting an estimated 21 million adults in the United States alone. A significant portion, approximately one-third, of those affected do not experience sufficient relief from standard oral antidepressant medications, leading to persistent symptoms that severely impact daily functioning and overall quality of life. TRD, a subset of MDD, contributes substantially to the economic burden associated with the disorder, accounting for nearly half of the total costs [5,6]. In the Indian context, data from the National Mental Health Survey 2015-16 indicated that around 15% of Indian adults require active intervention for mental health concerns, with approximately one in every twenty individuals suffering from depression. Alarmingly, in 2012, India recorded over 258,000 suicide deaths, predominantly affecting individuals aged 15 to 49 years. Globally, depression is recognized as the leading cause of disability, responsible for 7.5% of all years lived with disability as of 2015. The condition poses a particularly grave risk among youth, ranking as the second leading cause of death among those aged 15 to 29 years. At its most severe, depression can result in suicide, with more than 800,000 lives lost each year worldwide. In the Indian context, the National Mental Health Survey 2015-16 reported that around 15% of adults required active intervention for mental health concerns, with approximately one in 20 individuals suffering from depression. Suicide remains one of the most severe outcomes of untreated depression. According to the National Crime Records Bureau (NCRB) 2022, India recorded nearly 171,000 suicides, representing a suicide rate of 12.4 per 100,000 population, the highest documented to date. Notably, individuals aged 18-45 years accounted for almost two-thirds of these deaths, underscoring the disproportionate impact of suicidality on the most productive segment of the population [7].

At a biological level, TRD arises from multiple overlapping disturbances. Key mechanisms include glutamatergic system imbalance, hypothalamic-pituitary-adrenal (HPA) axis overactivity with sustained cortisol release, and chronic neuroinflammation that disrupts neurotransmission and neural repair. Reduced neuroplasticity, lower levels of brain-derived neurotrophic factor (BDNF), mitochondrial dysfunction, and oxidative stress further contribute to persistent symptoms beyond monoamine abnormalities. Together, these factors establish TRD as a distinct condition with complex neurobiology, as extensively reviewed by Kajumba et al. [8]. The extensive research efforts over the past decades have not yielded much success, and the currently used first-line conventional antidepressants are still ineffective for close to 66% of patients [9].

## Review

Pharmacotherapeutics

Guideline-Based Recommendations

The Indian Psychiatric Society (2017) and the American Psychological Association (APA, 2025) both recommend that TRD should be managed through a multifaceted and individualized approach. These guidelines emphasize systematic reassessment of diagnosis, optimization of antidepressant trials, and the considered use of augmentation, combination pharmacotherapy, or somatic interventions in a structured, stepwise manner [10].

Clinical Practice Realities

In day-to-day clinical practice, however, these recommendations are often adapted to patient-specific circumstances. Factors such as comorbid conditions, treatment history, medication access, and socioeconomic constraints strongly influence decision-making. Consequently, clinicians may rely more heavily on pharmacological augmentation strategies (e.g., lithium or second-generation antipsychotics) while access to neuromodulatory therapies such as repetitive transcranial magnetic stimulation (rTMS) or vagus nerve stimulation (VNS) remains limited to specialized centers [11].

Pharmacological augmentation remains a cornerstone of TRD management. Lithium augmentation, particularly effective in unipolar depression, has demonstrated efficacy in approximately 40% to 50% of patients, while remission is typically achieved in about 25% to 30%. Its benefits often become apparent within days to a few weeks and can be sustained throughout the acute phase of treatment [12].

Combination antidepressant therapy is also commonly employed when monotherapy proves insufficient. This involves using two agents with different mechanisms of action to potentially enhance therapeutic outcomes. For example, selective serotonin reuptake inhibitors (SSRIs) can be paired with tricyclic antidepressants (TCAs), although this necessitates careful monitoring due to possible pharmacokinetic interactions that can elevate toxicity risk [13].

Shelton et al. evaluated the olanzapine/fluoxetine combination in TRD. They observed a more rapid onset of antidepressant response compared with monotherapies, although by the study endpoint, efficacy did not differ significantly. The authors also noted methodological limitations, including patient entry criteria and randomization, and recommended improvements for future trials [14].

Electroconvulsive therapy (ECT) is widely regarded as one of the most effective interventions for severe or treatment-resistant depression. Systematic reviews and cost-effectiveness analyses have consistently demonstrated its therapeutic benefit and favorable economic profile, particularly in patients with high symptom burden or suicidal risk [15]. Beyond its clinical efficacy, ECT continues to be an important option in cases where rapid improvement is needed, such as severe depression with psychotic features or acute suicidality. In addition, recent consensus guidelines, including those from the National Network of Depression Centers, have emphasized the importance of harmonizing ECT documentation to ensure standardized practice and facilitate outcome monitoring across centers [15]. Together, these findings highlight ECT’s role as a well-established treatment while underscoring the need for consistent clinical reporting and quality improvement initiatives [15]. rTMS, which targets the left dorsolateral prefrontal cortex non-invasively, has demonstrated moderate efficacy and is generally well tolerated, although individual responses may vary [16]. Lastly, VNS is reserved for patients with more advanced, multi-treatment-resistant depression, particularly those who have not responded to four or more antidepressant regimens. Approved by the FDA for this subset, VNS has shown promise in maintaining long-term remission [17].

Ketamine and its derivative, esketamine, have emerged as innovative options in TRD. Intravenous (IV) ketamine, an N-methyl-D-aspartate (NMDA) receptor antagonist, is known for its rapid antidepressant effects, including marked reductions in suicidal ideation, even following a single infusion. Its mechanism, which modulates glutamate transmission, differentiates it from traditional treatments targeting monoamine systems [18]. Meanwhile, intranasal esketamine offers a more practical and convenient route of administration. Used in conjunction with oral antidepressants, it has shown prompt and sustained efficacy in alleviating depressive symptoms. Long-term data now support its safety and tolerability for extended use, reinforcing its role in chronic TRD management [19-21].

Esketamine

Mechanism of Action

**Figure 1:**
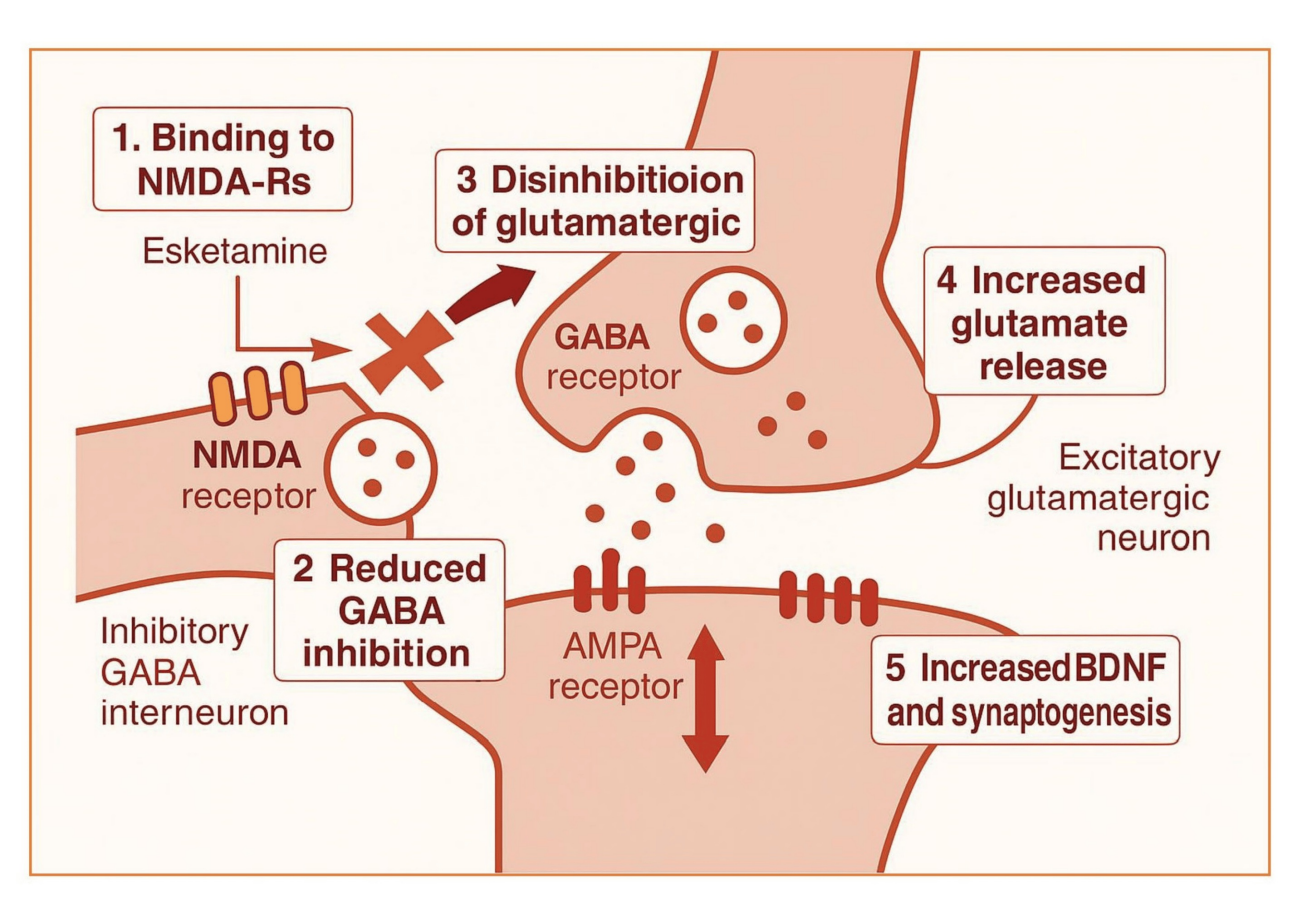
Mechanism of action of esketamine NMDA-Rs: N-methyl-D-aspartate receptors; GABA: gamma-aminobutyric acid; AMPA: α-amino-3-hydroxy-5-methyl-4-isoxazole propionic acid; BDNF: brain-derived neurotrophic factor This figure has been created by the author B. with Biorender.com.

Esketamine, the S-enantiomer of ketamine, exerts its antidepressant effects primarily through non-selective, noncompetitive antagonism of NMDA receptors, a subtype of ionotropic glutamate receptors. By blocking these receptors, esketamine indirectly triggers a temporary surge in glutamate levels, which in turn stimulates α-amino-3-hydroxy-5-methyl-4-isoxazole propionic acid (AMPA) receptors. This cascade enhances neurotrophic signalling and promotes synaptogenesis in brain regions responsible for mood regulation and emotional processing. Additionally, esketamine is believed to restore dopaminergic activity in motivation and reward-related brain circuits, contributing to its rapid therapeutic effects, such as alleviating anhedonia [22]. Its fast onset may also involve activation of the mammalian target of rapamycin complex 1 (mTORC1) pathway, which plays a key role in protein synthesis and supports brain-derived neurotrophic factor (BDNF) production, further enhancing synaptic connectivity and plasticity [23]. Figure 1 shows the mechanism of action of esketamine. This schematic is included for explanatory purposes and is not derived from a single clinical trial.

Dosage Forms and Strengths

Each intranasal spray device delivers a total dose of 28 mg of esketamine, administered via two sprays. The dosing schedule for intranasal esketamine in the treatment of TRD is structured into two phases: induction and maintenance. During the induction phase, spanning Weeks 1 to 4, esketamine is administered twice weekly, starting with a 56 mg dose on Day 1, followed by subsequent doses of either 56 mg or 84 mg based on the patient's response and tolerability. In the maintenance phase, the dosing is adjusted according to the patient's clinical needs. From Weeks 5 to 8, esketamine is administered once weekly at a dose of either 56 mg or 84 mg. Beginning in Week 9 and beyond, the frequency can be reduced to once every two weeks or maintained at once weekly, with doses continuing at 56 mg or 84 mg [24].

Clinical Evidences

In March 2019, the U.S. FDA approved Spravato (esketamine) nasal spray for use in combination with an oral antidepressant in adults with depression who had not responded to previous therapies [25]. Since then, multiple systematic reviews and meta-analyses have generally supported its efficacy and safety. For example, a meta-analysis by Fountoulakis et al. reported that although most randomized controlled trials conducted between Weeks 2 and 4 yielded negative or inconclusive results, the pooled analysis demonstrated a small but statistically significant reduction in depressive symptoms (effect sizes 0.15-0.23), comparable to outcomes typically seen with atypical antipsychotic augmentation in TRD [26]. Similarly, a 2024 meta-analysis comparing IV ketamine and intranasal esketamine across 12 studies found both routes to be effective, with IV ketamine showing significant benefit at 0.5 mg/kg and intranasal esketamine producing optimal outcomes at doses above 28 mg, particularly in the 56-84 mg range [27].

In January 2025, the U.S. FDA granted priority review approval for esketamine nasal spray as a standalone treatment for adults with TRD [28]. This decision, which permits use without concurrent oral antidepressants, was supported by a randomized, double-blind, placebo-controlled trial showing that esketamine monotherapy produced rapid and significant symptom improvement. Patients receiving esketamine achieved greater reductions in Montgomery-Åsberg Depression Rating Scale (MADRS) scores than those on placebo, with noticeable relief evident within 24 hours of the first dose. By Day 28, a post-hoc analysis indicated numerical improvements across all 10 MADRS items, and remission rates were higher with esketamine (22.5%) compared to placebo (7.6%) [29]. Importantly, the safety profile of esketamine monotherapy was consistent with previous adjunctive studies, with no new adverse effects observed. This expanded approval provides clinicians with greater flexibility to individualize therapy, offering the potential for rapid relief in patients with TRD without the need for daily oral antidepressants [28].

Review

Search Strategy 

In light of the evolving evidence on esketamine as a monotherapy for TRD, a comprehensive synthesis of current research is essential to guide future studies and therapeutic applications. A systematic search was conducted through March 2025 using major electronic databases such as PubMed and Google Scholar for published studies and medRxiv and bioRxiv for preprints. Manual searches of reference lists from relevant articles were also performed. The search strategy incorporated keywords like "esketamine," "inhalational," "monotherapy," "treatment-resistant depression," "clinical," and "randomized clinical trial," using Boolean operators (AND/OR/NOT) to refine the results. Studies were eligible if they were clinical trials evaluating the safety and efficacy of inhalational esketamine as a standalone treatment for TRD, written in English, and available in full text. Exclusion criteria included non-human studies (such as animal or in vitro research), case reports, reviews, abstracts, conference papers, editorials, commentaries, and book chapters. Two independent reviewer teams screened the titles and abstracts according to these criteria. Search results were recorded in Microsoft Excel (Microsoft Corp., Redmond, WA), where duplicate entries were removed based on matching author names, titles, and publication years. Full-text reviews were then conducted to confirm final eligibility. Of the 590 articles identified, only one study met all inclusion criteria, limiting the possibility of conducting a systematic review and meta-analysis and highlighting a significant gap in current research.

**Figure 2:**
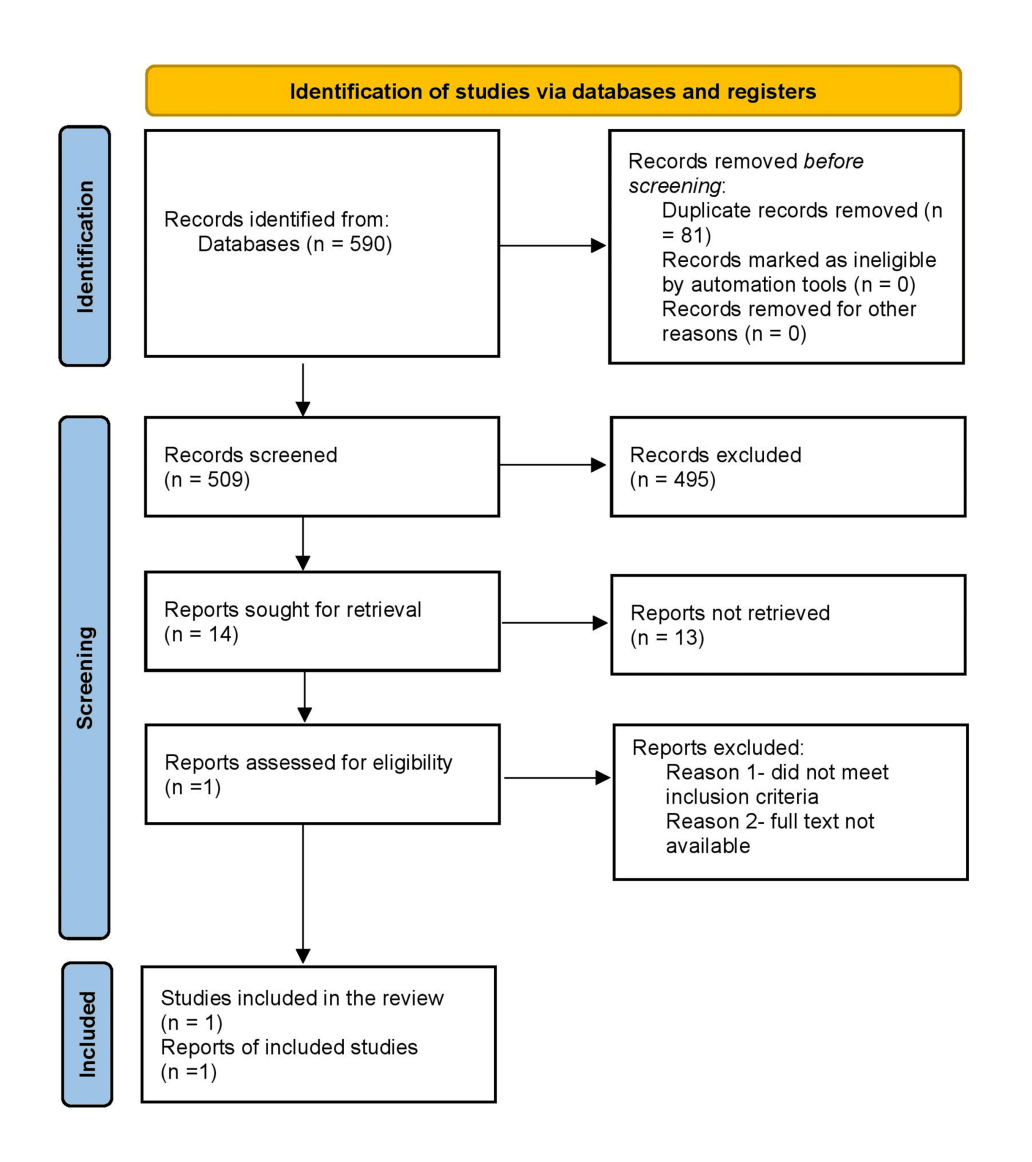
A PRISMA flowchart outlining the study selection process PRISMA: Preferred Reporting Items for Systematic Reviews and Meta-Analyses

Although only one eligible clinical trial was ultimately identified, the Preferred Reporting Items for Systematic Reviews and Meta-Analyses (PRISMA) flow chart was included to ensure transparency in reporting the search strategy and to demonstrate the rigorous screening process undertaken. This strengthens methodological clarity and adherence to systematic review reporting standards, even when evidence is limited. Figure 2 shows the PRISMA flow chart. 

This review, therefore, relies on the sole eligible SYNAPSE trial (NCT01998958) [30]. While this precluded a meta-analysis, documenting and critically appraising this single pivotal study highlights the current evidence gap and underscores the need for further high-quality research.

The sole eligible study, registered as NCT01998958 and referred to as the SYNAPSE study, was a randomized, double-blind, placebo-controlled, multicenter trial designed to evaluate the efficacy and safety of intranasal esketamine in adults with TRD [30]. Conducted across 25 sites in the United States, Japan, and Belgium, the study recruited participants aged 20 to 64 who met the Diagnostic and Statistical Manual of Mental Disorders, Fourth Edition (DSM-IV) criteria for MDD without psychotic features and who had failed to respond to at least two previous antidepressant treatments during their current depressive episode. The study was divided into two panels: Panel A included treatment arms administering 28 mg, 56 mg, or 84 mg doses of intranasal esketamine on Days 1, 4, 8, and 11, while the control group received a placebo with the possibility of re-randomization based on treatment response. Panel B evaluated lower-dose arms (14 mg or 56 mg) alongside a placebo group with similar re-randomization protocols. An optional open-label phase began on Day 15, during which all participants received 56 mg of esketamine, with dose adjustments permitted based on clinical response and tolerability. The primary outcome measured was the change in the MADRS total score by Day 28. Results showed a significantly greater reduction in depressive symptoms among esketamine-treated patients compared to those on placebo, with a least squares mean difference of -4.0 (SE 1.69; 95% CI: -7.31 to -0.64; one-sided p=0.010). By Day 28, 69.3% of patients in the esketamine group achieved a 50% or greater reduction in MADRS scores, compared to 52% in the placebo group. Additionally, remission rates (MADRS ≤12) were higher in the esketamine group at 52.5% versus 31.0% in the placebo group. Common adverse effects included nausea, dizziness, dissociation, unpleasant taste, headache, and vomiting. These findings support the rapid antidepressant effects and favorable safety profile of intranasal esketamine monotherapy in adults with TRD [30].

## Conclusions

Despite promising results, the available evidence for esketamine monotherapy in TRD remains limited. The SYNAPSE trial demonstrated rapid antidepressant effects and a favorable safety profile, but several trial-specific limitations, such as its short duration, open-label design after Day 15, exclusion of patients with psychotic or comorbid conditions, and reliance on MADRS scores alone, restrict the generalizability of findings. Broader concerns also persist regarding long-term safety, real-world applicability, and the potential for misuse or diversion, given ketamine’s established abuse liability. Although regulatory safeguards such as restricted distribution programs and observation periods are in place, robust data on adherence and long-term outcomes outside controlled trial environments are still lacking. These considerations underscore the urgent need for larger, longer-term, and more inclusive studies to better define esketamine’s true therapeutic potential, establish its long-term safety, and clarify its role in real-world clinical practice.
